# A Systematic Treatise, Historical, Etiological, and Practical, on the Principal Diseases of the Interior Valley of North America, as They Appear in the Caucasian, African, Indian and Esquimaux Varieties of Its Population

**Published:** 1850-06

**Authors:** 


*A Systematic treatise, Historical, Etiological, and Practical, on the principal diseases of the interior valley of North America, as they appear in the Caucasian, African, Indian and Esquimaux varieties of its population.* By DANIEL DRAKE, M. D. Cincinnati, Winthrop B. Smith & Co., publishers, Philadelphia; Grigg, Elliott & Co; New York, Mason & Sand, 1850. 8 vo. pp. 878.

This large volume is the first of a series, in the collection of materials for which the distinguished author has been many years engaged. The general character of the enterprise is expressed by the title-page, but to appreciate fully the comprehensiveness of its scope, and its specific objects, it is necessary to examine the work. By the expression "interior valley of North America," is designated an area estimated to embrace about six millions of square miles, constituting three fourths of the entire continental surface, beginning within the tropics, and terminating within the polar circle; bounded on the south by the Gulf of Mexico, on the north by the Polar Sea and Hudson Bay, on the east by the Appalachian, and on the west by the Rocky Mountains. After noting the prominent aspects of this vast territory, sketching its hydrographical features, &c., the seas, lakes, rivers and valleys which it embraces, and describing its geological outlines, the author divides the great interior valley for convenience of topographical description, into four hydrographical portions :—1st. the southern or Mexican ; 2d. the Eastern Lake, or St. Lawrence ; 3d. the Hudson ; 4th. the Arctic or Polar. The greater portion of the volume is devoted to investigations of the various conditions, geological, hydrographical, topographical, climatic, social and physiological—of these four divisions, and of the diseases standing in relation to these conditions. The purpose of the author is to study man in connection with the physical circumstances, and the moral influences appertaining to geographical situation, within the limits of the interior valley of North America, and to ascertain to what extent. and in what mode, the diseases to which he is subject are produced, or modified by these circumstances and influences. In so far, the inquiry is historical and etiological. It also enters into the plan of the author to consider, at length, individual diseases, discussing their causes, diagnosis, pathology, and treatment, About one hundred and sixty pages of the present volume are occupied with the consideration of periodical fevers, which the author prefers to designate *Autumnal* fevers.

It will be pereeived, if we have succeeded in briefly explaining the plan of the work, that the design is at once grand and philosophical. There are few men of sufficient hardihood to commence such an undertaking, and still fewer of sufficient perseverance to carry on successfully the labors which its prosecution has involved. A large share of the facts collected by Dr. DRAKE, have been obtained by personal explorations—visiting the prominent points in the vast territory over which the survey extends, examining for himself, and gathering details by communication with responsible individuals. Years have been consumed, and thousands of miles annually traversed in pursuit of the vast collection of data which he has now presented to the medical public. To obtain these data, and to arrange them for the induction of truth, requires something more than hardihood and perseverance, viz.—discrimination, judgment, knowledge. If we mistake not, the work will show, what before would not be denied, that the author possesses those higher qualifications, without which energy and industry were of little avail. Not professing to have given so voluminous a work a thorough, careful perusal, much of it being designed for reference, and of special interest only to persons residing in other portions of the Continent—still less, assuming competency to judge of its merits, or demerits, in many particulars, we do not hesitate to avow our belief that its publication is an event of no small importance in the progress of our national medical literature, and one which will make the name of DRAKE ever memorable in the history of American medicine. As a native production, we hail its appearance with gratification and pride. We trust our brethren over the Union will evince their appreciation of the undertaking by promptly exhausting the edition of this volume, and, in that way, encourage the continuance of labors, for which, and for the satisfaction of seeing the work completed, we sincerely hope, the life and health of the distinguished author may be spared.

